# Editorial: Challenging dogma: evolution in endourological techniques and management

**DOI:** 10.3389/fruro.2023.1226476

**Published:** 2023-07-11

**Authors:** Wesley Yip, Amir Lebastchi, Saum Ghodoussipour

**Affiliations:** ^1^ Urology Service, Department of Surgery, Memorial Sloan Kettering Cancer Center, New York, NY, United States; ^2^ USC Institute of Urology, University of Southern California, Los Angeles, CA, United States; ^3^ Department of Urology, Rutgers Robert Wood Johnson University Hospital, New Brunswick, NJ, United States

**Keywords:** endourology, HOLEP, holmium laser enucleation of the prostate, robotic surgery, ureteroscope

Urology is one of the most innovative surgical fields, but many dogmas continue to exist. Without appropriate challenge and discourse, these dogmas will perpetuate based on eminence rather than evidence. While the merits of certain approaches have become commonplace, such as ureteroscopy in lieu of open stone extraction, other debates rage on, including robotic versus open surgery. Indeed, the field of endourology was created from the desire to innovate and disseminate minimally invasive procedures with less morbidity but equal effectiveness. In line with the scope of Frontiers in Urology, which encourages novel research that challenges older existing principles, we organized this Research Topic to provide researchers the opportunity to challenge these dogmas and engage in robust academic discourse.

The first article by Kronstedt et al., “The role of bowel for minimally invasive treatment of stricture disease” explores the various approaches to reconstruction of the urinary tract, from traditional open techniques, to difficult laparoscopic methods, to finding a balance between function and recovery with robotic assistance. While ileum has been utilized for upper tract reconstruction for over a century, there has been increasing usage of the appendix, especially on the right side, as either an interposition or an onlay graft. In the lower tract, buccal mucosa has been challenged by rectal mucosa harvests, which can be obtained transanally and reduce morbidity and enhance recovery in these patients.

In another article exploring stricture management, “Allium stent as a curative treatment for benign ureteral strictures: Preliminary experience, surgical technique, and functional results”, Salciccia et al. report on the efficacy of dilation and temporary metal stent placement in lieu of chronic stent exchanges or invasive surgery and reconstruction. In an evaluation of 83 patients with a median stricture length of 2.5cm, their success rate with a median follow-up of 18 months is quite high – certainly worth consideration, especially since the stent is able to be removed. We eagerly await an update in the future in regards to long-term durability.

In terms of BPH management, Ganesh et al. question “Effects of 5-alpha reductase inhibitors prior to Holmium Laser Enucleation of the prostate: Does increased adenoma density result in prolonged morcellation times?”. As HoLEP becomes increasingly popular, and many of these men have suffered from BPH for an extended period of time and failed medical management, i.e. with 5-alpha reductase inhibitors, this question becomes quite salient for the endourologist and surgical planning. They perform a matched analysis in over 300 patients from an expert center to provide valuable data to the field.

Moving from benign to malignant, Chien et al. provide a thorough review of “The role of endoscopic management and adjuvant topical therapy for upper tract urothelial cancer”. Given the significant morbidity and renal function loss with radical nephroureterectomy, percutaneous and ureteroscopic options are appealing in an often difficult to treat population. The disease is notoriously difficult to appropriately diagnose and stage, but numerous enhanced evaluation methods have emerged, including endoluminal ultrasonography, narrow-band imaging, photodynamic diagnosis, optical coherence tomography, and diagnostic confocal laser endomicroscopy. Tumor destruction methods are also advancing, with neodymium:yttrium-alumnium-garnet (YAG), holmium:YAG, and thulium:YAG lasers, cryoablation, and chemo/immunotherapeutic instillations.

Lastly, Di Gianfrancesco et al. ask the question, “Could HoLEP change the further management of incidental prostate cancer?”. Similar to incidental prostate cancer diagnosis at the time of transurethral resection of the prostate for presumed BPH, a subset of HoLEP patients will be newly diagnosed with prostate cancer. In this study, the oncologic outcomes of patients with prostate cancer diagnosis prior to, or as a result of, HoLEP are compared.

The goal of this Research Topic was to provide a forum in which authors could present original endourological research that helps to push the whole urological field forward, or at least provide data that can be debated. Many practice patterns are resistant to change, since stalwarts in the field have championed a technique for decades. Certain methods withstand the test of time, while others are replaced. We feel that these articles do indeed help to push the field forward, by providing food for thought and fodder for discussion.

## Author contributions

All authors listed have made a substantial, direct, and intellectual contribution to the work and approved it for publication.

